# The Influence of Nordic Walking Training on the Serum Levels of Sirtuins, FOXO3a, and Vitamin D Metabolites in Patients with Multiple Myeloma

**DOI:** 10.3390/curroncol31120587

**Published:** 2024-12-14

**Authors:** Olga Czerwińska-Ledwig, Małgorzata Żychowska, Artur Jurczyszyn, Joanna Kryst, Adrianna Dzidek, Roxana Zuziak, Anna Jurczyszyn, Anna Piotrowska

**Affiliations:** 1 Institute for Basic Sciences, Faculty of Physiotherapy, University of Physical Education, 31-571 Krakow, Poland; joanna.kryst@awf.krakow.pl (J.K.); roxana.zuziak@awf.krakow.pl (R.Z.); 2 Faculty of Health Sciences and Physical Culture, Biological Fundation of Physical Culture, Kazimierz Wielki University in Bydgoszcz, 85-064 Bydgoszcz, Poland; 3 Plasma Cell Dyscrasia Center, Department of Hematology, Faculty of Medicine, Jagiellonian University Medical College, 31-501 Krakow, Poland; 4 Faculty of Medicine, Jagiellonian University Medical College, 31-501 Krakow, Poland; anna.jurczyszyn@student.uj.edu.pl

**Keywords:** multiple myeloma, physical activity, nordic walking, sirtuins, FOXO3a, vitamin D metabolites

## Abstract

Background: Multiple myeloma, a malignancy of plasma cells, often involves the disruption of vitamin D metabolism. Vitamin D, acting through its receptor (VDR), affects transcription factors like FOXO and sirtuins, which regulate cellular processes. The impact of physical activity on these markers in multiple myeloma patients is unclear. Therefore, the objective of this study was to evaluate the effects of a 6-week training program on these parameters. Material and methods: The study was completed by 30 patients, including 16 in the Nordic walking training group (TG) and 14 in the control group (non-exercising, CG). All participants underwent a thorough medical interview before starting the project. Venous blood samples were collected from all participants four times—at baseline, after 3 weeks, after 6 weeks, and after 9 weeks (follow-up). The serum concentrations of sirtuin 1, sirtuin 3, Foxo3a, vitamin D receptor (VDR), 25(OH)D3, and 1,25(OH)2D were determined. Body composition, physical fitness, and physical activity level were assessed at baseline and after 6 weeks. Results: No statistically significant changes were observed in the serum levels of sirtuins, the FOXO3a protein, and 1,25(OH)2D. A statistically significant difference was observed in the levels of VDR for both time and group factors, but this was not confirmed in the post hoc test. Vitamin 25(OH)D3 level increased significantly in the study group with time. Conclusions: The applied 6-week Nordic walking training cycle positively affected the level of vitamin 25(OH)D3 but did not influence the rest of the biochemical parameters studied. The obtained results also indicate that the applied intervention is safe for patients and does not interfere with body composition.

## 1. Introduction

Multiple Myeloma (MM) constitutes approximately 10–15% of all diagnosed hematologic malignancies and around 1–2% of all cancer cases [1], primarily affecting elderly individuals, with a median age at diagnosis of 70 years [2]. This disease arises from the clonal expansion of plasma cells within the bone marrow, which, following neoplastic transformation, synthesize altered immunoglobulins referred to as monoclonal proteins. The production of these proteins, coupled with the activity of neoplastic cells, results in a range of organ-specific complications that form the basis for diagnosing MM. The diagnostic criteria include elevated serum calcium level (C), renal insufficiency (R), anemia (A), and the presence of osteolytic lesions in the bones (B). Additionally, as expanded by the SLiM criteria, there is a percentage of plasma cells in bone marrow biopsy of at least 60% (S—sixty), the presence of clonal light chains (Li), and the presence of bone infiltration detected by magnetic resonance imaging (M). In the case of fully symptomatic MM, treatment aims to achieve disease remission. For individuals under 70 years of age in good general condition, qualification for autologous stem cell transplantation is conducted, preceded by induction therapy and followed by myeloablative therapy [3]. Patients who are not eligible for transplantation are treated with chemotherapy protocols.

Vitamin D, due to its mode of action in the body, is currently classified as a hormone with pleiotropic effects [4]. Its significant association with the development of civilization diseases, including cancer, has been indicated [5]. When ingested through food, the inactive form of vitamin D undergoes hydroxylation in the liver to form calcidiol 25(OH)D3. Further hydroxylation occurs in the proximal tubules of the kidneys, resulting in the formation of 1,25(OH)_2_D (calcitriol). Calcitriol is the active form that affects gene expression in target tissues by binding to the receptor (VDR), regulating processes associated with cell proliferation and differentiation. It has been shown that target genes regulated by VDR ligands overlap with genes regulated by the FOXO3a protein (Forhead box). VDR interacts with FoxO proteins and their regulator, sirtuin 1 (SIRT1), and 1,25(OH)_2_D induces the SIRT1-dependent dephosphorylation and activation of FoxO protein functions [6]. Many studies indicate the dysregulation of vitamin D metabolism and function in various cancers [7]. On the other hand, the significance of vitamin D in physical activity is well established [8]. Physical training, such as Nordic walking has a beneficial effect on serum calcidiol levels, as demonstrated previously in a study involving perimenopausal women [9] and patients with multiple myeloma [10].

FOXO proteins are nuclear transcription factors that regulate many cellular processes related to apoptosis and the cell cycle. This family consists of the proteins FOXO1, FOXO3a, FOXO4, and FOXO6 [11]. The suppression of the FOXO3a protein has been shown to be associated with increased inflammation and the development of autoimmune diseases through the activation of the nuclear transcription factor NF-κB [12]. The activation of the NF-κB-related pathway leads to the increased synthesis of cytokines, chemokines, growth factors, acute-phase proteins, adhesive molecules, and anti-apoptotic proteins, and it also enhances the expression of genes that inhibit the accumulation of reactive oxygen species [13]. Some treatment methods for hematologic malignancies have been shown to positively influence the activity of FOXO3a protein. Hypomethylating agents increase FOXO3a activity in acute myeloid leukemia [14]. In Hodgkin’s lymphoma, supplementation with resveratrol led to cell apoptosis associated with SIRT1 inhibition and increased FOXO3a acetylation [15]. Sirtuins (SIRT) are enzymes belonging to the class III NAD^+^-dependent histone deacetylases. In mammals, there are seven types of these proteins (from SIRT1 to SIRT7). They participate in post-translational protein modification, playing a role in various metabolic processes [16]. These proteins are also considered significant in cancer. Depending on the type of tumor, SIRT1 can have either a suppressive or oncogenic role [17]. An increased expression of *SIRT1* was observed in myeloma cells lines and may be associated with treatment resistance [16]. While the expression of *SIRT 2* and *SIRT3* in MM patients was found to be lower than in healthy controls [18]. Physical exercise of various types and intensities can affect SIRT 1 [19] and SIRT 3 serum levels [20]. The direction of these changes depends on multiple factors, including the type, duration, and intensity of the activity, as well as the physiological characteristics of the study’s participants.

Several beneficial effects of regular physical activity in cancer patients have already been indicated in the available literature. However, this specific group needs a well-chosen, safe form of training. Marching training is often recommended for this purpose. Additional enrichment of walking through the use of poles allows the enhancement of training effects and increases body stability [21,22]. Nordic walking, a form of walking with poles, is considered a safe and beneficial physical activity, recommended for both older adults and patients with hematologic malignancies [21,23]. Training conducted in older individuals may have a positive impact on body composition as well as functional fitness. However, there is a lack of research examining the effects of this type of physical activity on the serum levels of vitamin D metabolites, SIRT1, and FOXO3a proteins. Previous studies have indicated the safety of Nordic walking training in patients with MM. The importance of the aforementioned parameters in multiple myeloma, along with the well-documented effects of physical activity on their modulation, has motivated the authors to pursue the proposed research topic.

The aim of this study was to investigate the effects of a 6-week cycle of Nordic walking training intervention on the serum levels of vitamin D metabolites (25-OH-D3 and 1,25-OH-D3) and vitamin D receptors VDR, SIRT1, SIRT 3, and FOXO3a in patients with multiple myeloma. Employing an interventional research design with the assessment of biochemical marker levels at various points during the training cycle, the study aimed to investigate the impact of the intervention on the dynamics of change in these markers. Based on a review of the literature, we hypothesized that the training intervention may induce changes in the examined sirtuins. In the case of vitamin D metabolites, these changes were expected to be beneficial. Additionally, we anticipated that changes in body composition and functional fitness could also occur; however, with moderate-intensity exercise, these effects would not be pronounced.

## 2. Materials and Methods

### 2.1. Study Group

**Figure 1:**
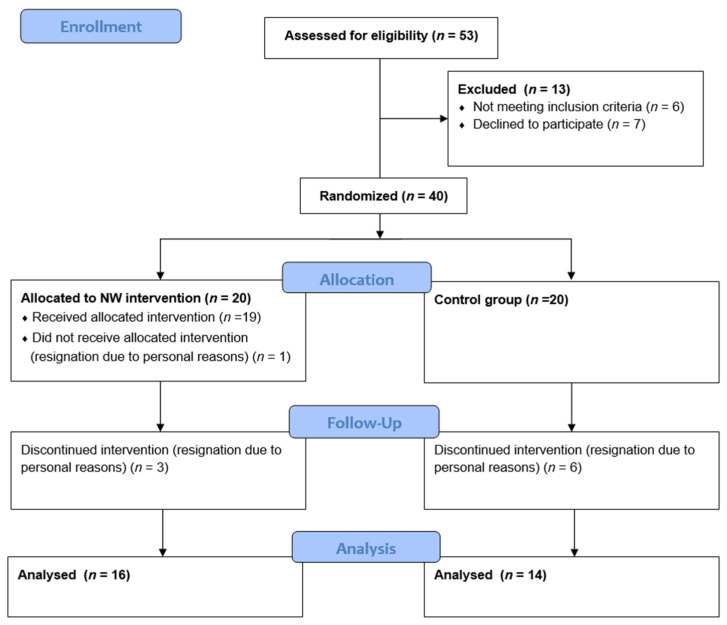
CONSORT patient flow diagram.

The study included 40 patients with MM, recruited from the Department of Hematology at Jagiellonian University Medical College in Krakow. Participants were randomly assigned to one of the following two groups: those undergoing Nordic walking health training (TG, *n* = 20) and a control group—non-exercising (CG, n = 20). For the random group assignment, a permuted block randomization procedure (1:1 ratio) was employed. Each participant selected an opaque envelope containing their group allocation. The flowchart of patients’ enrollment is included in Figure 1.

Inclusion in the study required a thorough medical interview and meeting the inclusion criteria presented in Table 1. The participants were extensively informed about the study protocol and the option to withdraw consent to participate in the project at any stage without consequences.

### 2.2. Study Protocol

Before allocation to groups, each participant underwent the following procedures:

1. Medical interview and examination (including questionnaires, body composition analysis, anthropometric measurements, and aerobic capacity assessment);
2. Venous blood collection for biochemical analyses;
3. Randomized assignment to one of the following two groups, training group (TG) or control group (CG).

Blood samples were collected from patients in both groups at the following four times: before the start of the project (baseline, I), after 3 weeks (II), after 6 weeks (III), and after 9 weeks from the start of participation in the project (follow-up, IV). The body composition analysis and the aerobic fitness assessment were performed before (I) and repeated after 6 weeks (immediately after the end of the training period, II).

### 2.3. Methods

#### 2.3.1. Interview—Questionnaires

In addition to the standard medical interview, before starting and after completing the training, an assessment of the physical activity level was conducted using the IPAQ questionnaire, short version [24], as well as a dietary analysis using a 3-day food diary based on the Food Photography Album [25].

#### 2.3.2. Body Composition Analysis, Anthropometric Measurements, and Aerobic Capacity Assessment

Body composition analysis was performed using the bioimpedance method (BIA) with the Tanita BC 418 MA scale (93/42 EEC, Tanita, Tokyo, Japan). The following variables were estimated: BMI—body mass index, FAT%—body fat percent, FFM—fat free mass, MM—muscle mass, TBW—total body water. The height, waist circumference, and hip circumference of the participants were also measured. Additionally, an assessment of the aerobic capacity was performed in all participants with the use of a 2 min step test proposed by Rikli and Jones [26]. All these parameters were assessed twice—before the start of the project and after 6 weeks.

#### 2.3.3. Venous Blood Collection

Venous blood was collected from all participants at the following 4 times: before the start of the project (I), after 3 weeks (II), after 6 weeks (III) and after 9 weeks (IV, follow-up). Blood drawing was performed by a phlebotomist using a vacuum system, with each sample collected into a tube with a clot activator. After clot formation, each sample was centrifuged at 2500 rpm for 10 min at 4 °C (MPW-351R, MPW, Warszawa, Poland). It was then transferred to Eppendorf tubes and stored at −80 °C in a low-temperature freezer until measurements were conducted. Serum concentrations of SIRT1, SIRT3, FOXO3a protein, vitamin D receptor (VDR), 25(OH)D3, and 1,25(OH)2D were determined using an enzyme-linked immunosorbent assay (ELISA) method, using commercially available kits with the appropriate sensitivity and specificity.

### 2.4. Nordic Walking Training

The 6-week health training program comprised 18 sessions, held three times a week in the morning, each lasting approximately 60 min. All sessions were conducted by a certified Nordic walking instructor and supervised by members of the research team. The program took place outdoors during the spring–summer period.

Each training unit included a 10 min warm-up (light exertion), a 45 min main part (moderate exertion), and a 5 min cool-down part (light exertion). During the main part, participants performed progressively prolonged Nordic walking with proper technique. The training intensity was individually adjusted for each participant with the use of the Nes formula [27] to a level corresponding to 60–70% of the calculated HR and monitored using a heart rate monitor (M400, Polar, Kempele, Finland).

### 2.5. Statistical Analysis

The sample size was calculated using the standard method for determining sample size for proportions with a finite population correction (FPC), considering a confidence level of 90%, a margin of error of 10%, and a proportion of 0.5, resulting in a minimum required sample size of 30 patients.

For all continuous variables, descriptive statistics were calculated and presented as the mean and standard deviation. The normality of distribution was assessed using the Shapiro–Wilk test. For dietary analysis, the independent sample t-test was used if the normality assumption was met; otherwise, the Mann–Whitney U test was performed as its non-parametric equivalent. For other variables with repeated measurements, repeated measures analysis of variance (RM ANOVA) was used. The assumptions of the tests (homogeneity of variances, Levene’s test; sphericity, Mauchly’s test) were checked for all variables. If the assumptions were not met, appropriate corrections or non-parametric tests (Friedman test) were applied. Post hoc analysis using the Bonferroni test was conducted for statistically significant results. All calculations were performed using JASP 0.16.4 software (University of Amsterdam, The Netherlands).

## 3. Results

### 3.1. Study Group Characteristics

A total of 30 participants completed the project, including 16 in the TG (9 females, 7 males; mean age: 62.3 ± 8.2 years) and 14 in the CG (7 females, 7 males; mean age: 63.6 ± 3.6 years). The dietary analysis results of the participants are presented in Table 2. There were no statistically significant differences in the content of individual nutrients between the two groups.

### 3.2. Anthropometric Measurements, Body Composition Analysis, and Physical Activity Level

The results are shown in Table 3. The physical activity level increased in the TG from 1345.34 ± 158.31 MET-min/week at baseline (I) to 2239.38 ± 246.67 MET-min/week after 6 weeks (III). In the control group, it changed from 1397.64 ± 182.00 MET-min/week to 1396.39 ± 196.92 MET-min/week. The observed change was statistically significant between the groups (*p* < 0.001, F = 39.209) as well as over time (*p* < 0.001, F = 149.707) and in the time×group interaction (*p* < 0.001, F = 15.546). Post hoc tests revealed statistically significant differences in the TG between measurements I and III (*p* < 0.001, t = −17.936). Additionally, significant differences were found between measurement II in the TG and measurements I and III in the CG (*p* < 0.001, t = −11.543; *p* < 0.001, t = −11.561).

For the 2 min step test, statistically significant differences were observed for the time of measurement (*p* = 0.002, F = 12.327) as well as in the group*time interaction (*p* = 0.010, F = 7.664). Post hoc tests confirmed that significant changes occurred in the TG. Statistically significant differences were identified for the TG at baseline vs. TG after 6 weeks (III, *p* < 0.001) and the CG vs. TG after 6 weeks (III, *p* = 0.022). The results close to statistical significance were shown for the CG at baseline vs. TG after 6 weeks (III, *p* = 0.056).

### 3.3. Serum Biochemical Parameters

For SIRT1 and Foxo3 proteins, no statistically significant differences were observed in the RMANOVA analysis. However, for SIRT3, a difference between groups was identified (*p* = 0.0277, F = 5.863). This finding was not confirmed by the post hoc tests.

**Figure 2:**
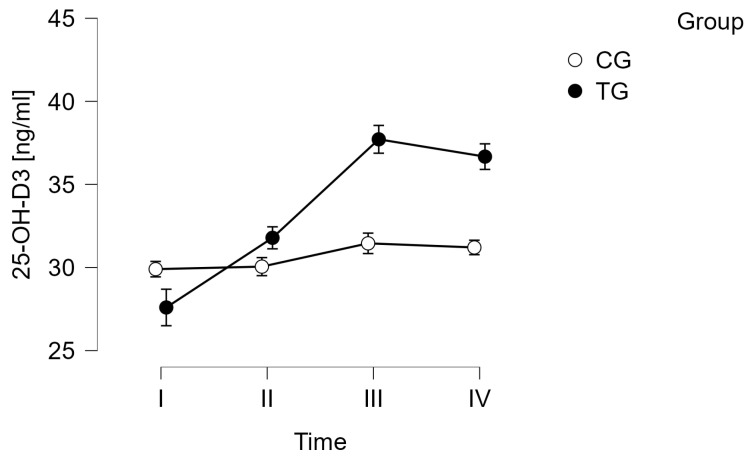
Serum levels of vitamin 25-OH-D3 in participants of the project. CG—control group; TG—training group.

In the case of vitamin 25-(OH)-D3, despite the absence of significant changes in VDR and 1,25-(OH)-D3 concentrations, statistically significant changes over time were observed (*p* < 0.001, F = 27.440). Statistical significance was also found for the group*time interaction (*p* < 0.001, F = 14.328). Observations were confirmed by post hoc tests. Significant changes were observed only in the training group (TG), where an increase in 25-(OH)-D3 concentration occurred at the following time points: I vs. II (*p* = 0.002), I vs. III (*p* < 0.001), I vs. IV (*p* < 0.001), as well as II vs. III (*p* < 0.001) and II vs. IV (*p* < 0.001). The results are shown in Table 4 and Figure 2.

## 4. Discussion

The applied 6-week Nordic walking training cycle caused a significant increase in the vitamin 25-OH-D3 serum level but did not cause significant changes in the serum levels of the studied proteins and vitamin 1,25-OH-D3. No changes in body composition were observed in either group, whereas in the training group, aerobic fitness significantly increased, as measured by the 2 min step test.

In the context of introducing new oncological therapies and the extended survival times of patients with cancer, including multiple myeloma, patient rehabilitation has become essential. Studies indicate that patients with hematological malignancies are encouraged to engage in physical activity at every stage of treatment, as well as after its completion [28]. However, it is crucial that the type of activity not only aids in maintaining muscle mass and functional fitness but is also safe from the perspective of the biochemical parameters related to the disease and the molecular-level changes. For patients with multiple myeloma, this issue has been explored particularly in relation to Nordic walking training, with prior documentation available on this topic [10,29,30].

The review of the available literature indicates that various forms of physical activity prescribed to patients with different hematologic cancers have a differentiated impact, particularly for those undergoing oncological therapy. Exercise is shown to improve quality of life from a small to a significant degree, and it helps reduce treatment-related side effects [31]. The most beneficial effects on functional fitness for patients undergoing oncological therapy are achieved through exercise programs combining aerobic and resistance training, particularly at moderate to vigorous intensity levels [32].

One key finding of this study is the improvement in aerobic capacity among participants, as confirmed by the 2 min walk test. This straightforward test demonstrates enhanced exercise tolerance, which, as research shows, is an essential component influencing quality of life. In this study, a significant improvement in aerobic capacity was observed as the physical activity levels of participants increased, as assessed by the 2 min walk test. Similar positive changes in patient endurance were reported in a study by Coleman et al., in which a 15-week exercise regimen yielded improved functional capacity [33]. However, their study was conducted with patients actively undergoing therapy, unlike our cohort, which consisted of patients in remission. In the study by Hacker et al., a physical activity monitoring program was implemented for a group of patients post-HCT transplantation, with specific daily activity goals set; their symptom changes and functional capacity were compared to a control group receiving standard care. After six weeks, both groups showed psychological improvements and reductions in perceived physical symptoms, although a decline in functional fitness was observed in both groups [34]. The intervention in our study also lasted six weeks, and the observed substantial improvement in functional fitness may be linked to the intensity of physical activity undertaken, as well as the fact that our participants were in remission rather than immediately post-hospital treatment. This suggests that six weeks of regular moderate-intensity training can yield noticeable endurance benefits. Notably, these changes occurred without alterations in body composition, which is advantageous for preserving muscle mass and maintaining energy balance, thereby preventing excessive weight loss. Increasing the intensity and duration of training could potentially lead to beneficial changes, such as a reduction in fat mass and an increase in muscle mass. Achieving safe weight reduction is of significant clinical importance [35].

In this study, no significant changes were observed in the serum concentrations of SIRT2, SIRT3, and FOXO3a proteins, indicating that participation in the training did not affect the balance of these proteins, which plays a role in regulating cellular processes such as cellular aging, oxidative stress resistance, and apoptosis. The impact of the lack of exercise on the concentrations of these proteins suggests that the training did not disrupt their serum levels. It should be noted that the role of sirtuin proteins in oncology patients may differ from their role in healthy individuals, as these proteins are considered critical in hematologic malignancies [36]. Reduced concentrations of SIRT1–3 have been observed in older adults with frailty syndrome, a condition often associated with cancer [37]. In patients with multiple myeloma, a reduction in SIRT3 levels alongside elevated clinical markers of inflammation may be significant in the development of treatment-related peripheral neuropathy [38]. Furthermore, decreased SIRT2 and SIRT3 concentrations have been correlated with disease progression and increased redox imbalance in multiple myeloma patients [18]. Sirtuin family proteins are closely linked to cellular oxidative stress, and fluctuations in their concentrations may reflect a cellular response to oxidative stress [39]. Previous studies have shown that Nordic walking exercise regimens in multiple myeloma patients did not affect the levels of oxidative damage to macromolecules [30], which may explain the lack of changes in serum protein concentrations observed in the present study. As shown by the control group, the concentrations of these parameters in the serum of patients in the plateau phase of MM remained at a relatively stable level.

Among the evaluated parameters related to vitamin D metabolism, a significant increase in vitamin 25-OH_-_D_3_ levels was observed in the training group relative to baseline as early as three weeks after starting the exercise regimen. This upward trend continued until the end of the training program, followed by a slight decrease in serum levels three weeks post-intervention (follow-up). Interestingly, there were no significant changes in the concentrations of 1,25-OH_-_D_3_ or VDR. Despite normal renal function parameters, which were a criterion for inclusion in the study, the conversion of vitamin 25-OH_-_D_3_ in the kidneys may be impaired in these patients. This may be related to the dysregulation of the enzymes responsible for this conversion. It is also possible that the exercise regimen in this study was not intense enough to significantly stimulate bone metabolism. However, when introducing physical activity for patients with multiple myeloma, who often experience bone-related complications, it is essential to maintain an exercise intensity that balances safety with the potential benefits. In a previous study involving a different patient cohort, Nordic walking did not affect serum phosphorus levels but contributed to an increase in vitamin 25-OH_-_D_3_ and calcium concentrations [10]. The similar changes vitaminin vitami_3_n 25(OH)D_3_ observed in the present study may be attributed to an increased release of vitamin D stored in adipose tissue. Vita_3_min 25(OH)D_3_ serves as a storage form of vitamin D in both obese individuals and those with normal body weight [40]. This effect may be associated with the increased metabolic activation of adipose tissue, as no reduction in fat percentage (%FAT) or fat mass was detected in the body composition analysis. This explanation can be supported by the observation that no significant changes in the measured parameters related to vitamin D metabolism occurred in the control group, which did not engage in physical activity.

### Study Strengths and Limitations

The primary strength of this study lies in its novelty, as the subject has not been previously explored in the existing literature. The results obtained indicate that the proposed training regimen has a beneficial and safe impact on MM patients regarding the studied parameters. However, a notable study limitation was the small sample size; therefore, further research should be conducted on a larger group of patients.

## 5. Conclusions

The 6-week cycle of Nordic walking training had a beneficial effect on the patients’ aerobic capacity. The observed increase in the serum concentration of v_3_itamin 25-OH-D_3_ is beneficial, in particular due to the fact that the baseline concentrations were relatively low, despite the standard supplementation with choleocalciferol, which was not changed during participation. The results obtained confirm the beneficial effect of the applied training on the patients. The results obtained also indicate that the intervention used is safe for patients and does not interfere with body composition, which is beneficial for oncological patients. The applied intervention is safe also in the context of the measured sirtuins and FOXO3a protein levels. Despite the absence of anticipated changes in these parameters, the training did not disrupt the balance of their concentrations in the serum, reflecting its beneficial effect. The proposed form of training can therefore be recommended to patients with MM as an effective way to maintain physical activity at an optimal level.

## Figures and Tables

**Table 1:** Inclusion and exclusion criteria.

Inclusion Criteria	Exclusion Criteria
Multiple myeloma in plateau stage without cytostatic treatment	Significant liver and kidney damage
Permissible bisphosphonate therapy	Acute respiratory tract infection or other infectious disease
Overall good condition of the patient	Another malignancy
Vitamin D and calcium supplementation in accordance with standards	Recent fall from one’s own height resulting in injury

**Table 2:** Diet analysis results (performed at baseline).

	TG (n = 16)	CG (n = 14)
	Mean ± SD	Mean ± SD
Energy [kcal]	2373.6 ± 305.6	2450.9 ± 310.5
Water [g]	2857.6 ± 730.7	3184.1 ± 777.8
Protein [g]	110.4 ± 23.0	97.1 ± 20.2
Fat [g]	90.8 ± 18.9	93.9 ± 31.0
Carbohydrates [g]	293.0 ± 68.6	328.8 ± 76.1
Vitamin A [μg]	1465.3 ± 876.0	1406.4 ± 768.1
Vitamin E [μg]	9.5 ± 4.1	9.1 ± 4.6
Vitamin C [mg]	100.6 ± 58.1	89.3 ± 46.9
Folic acid [μg]	343.9 ± 103.3	343.5 ± 54.8
Vitamin D [μg]	5.1 ± 3.6	5.4 ± 7.7
Energy from protein [%]	18.7 ± 3.0	16.0 ± 3.2
Energy from fat [%]	34.1 ± 6.7	34.0 ± 10.6
Energy from carbohydrates [%]	45.4 ± 6.4	50.1 ± 10.8

TG—Nordic walking group; CG—control group; SD—standard deviation. No statistically significant differences were observed between groups.

**Table 3:** Body composition and anthropometric measurements.

	TG (n = 16)			CG (n = 14)		
	Baseline (I) Mean ± SD	After 6 Weeks (III) Mean ± SD	Δ	Baseline Mean ± SD	After 6 Weeks (III) Mean ± SD	Δ
Body composition						
Body mass [kg]	78.72 ± 10.44	78.53 ± 10.57	−0.19	79.44 ± 15.56	79.91 ± 15.61	+0.47
BMI [kg/m]	29.29 ± 3.40	29.26 ± 3.40	−0.04	28.34 ± 4.28	28.48 ± 4.34	+0.14
FAT% [%]	34.40 ± 7.13	34.28 ± 7.32	−0.12	33.12 ± 7.47	32.79 ± 6.22	−0.33
FFM [kg]	51.52 ± 8.40	51.51 ± 8.43	−0.02	52.61 ± 9.84	55.44 ± 11.50	+2.83
MM [kg]	48.91 ± 8.02	48.89 ± 8.04	−0.02	49.95 ± 9.37	50.57 ± 9.65	+0.62
TBW [%]	45.82 ± 4.80	45.80 ± 4.82	−0.03	46.44 ± 4.66	47.05 ± 4.0	+0.61
Anthropometrics						
Body height [cm]	164.0 ± 10.2	-	-	166.9 ± 6.1	-	
Waist [cm]	94.32 ± 8.84	95.36 ± 9.22	+1.04	97.57 ± 15.66	97.87 ± 16.11	+0.30
Hips [cm]	104.23 ± 8.52	103.24 ± 7.76	−0.99	103.57 ± 10.87	103.40 ± 10.74	−0.17
Aerobic capacity						
2 min step test [repetitions]	96.4 ± 19.7 *	108.8 ± 14.0 *^,$^	+10.81	91.0 ± 18.3	92.5 ± 15.70 ^$^	+1.36

TG—Nordic walking group; CG—control group; Δ—mean change; SD—standard deviation. BMI—body mass index; FAT%—body fat percent; FFM—fat free mass; MM—muscle mass; TBW—total body water. *—statistically significant changes in TG; ^ $^—statistically significant changes between the CG and TG.

**Table 4:** Results of serum biochemical parameter measurements.

		TG (n = 16)	CG (n = 14)			TG (n = 16)	CG (n = 14)
		Mean ± SD	Mean ± SD			Mean ± SD	Mean ± SD
Sirt1 [ng/mL]	I	4.12 ± 0.16	4.13 ± 0.19	25-(OH)-D3 [ng/mL]	I	27.60 ± 12.00 *	29.90 ± 7.46
	II	4.21 ± 0.09	4.19 ± 0.17		II	31.78 ± 10.56 *^,#,$^	30.05 ± 6.53
	III	4.19 ± 0.07	4.30 ± 0.17		III	37.71 ± 11.28 *^,#^	31.45 ± 7.77
	IV	4.19 ± 0.12	4.28 ± 0.16		IV	36.67 ± 10.60 *^,$^	31.20 ± 6.79
Sirt3 [ng/mL]	I	4.97 ± 0.39	5.18 ± 0.35	1,25-(OH)-D3 [ng/mL]	I	205.42 ± 17.72	207.19 ± 20.28
	II	5.16 ± 0.21	5.21 ± 0.21		II	210.82 ± 8.01	211.39 ± 6.41
	III	5.06 ± 0.17	5.08 ± 0.65		III	210.65 ± 4.24	209.34 ± 16.41
	IV	5.16 ± 0.18	5.37 ± 0.28		IV	213.85 ± 6.15	208.76 ± 5.89
Foxo3 [ng/mL]	I	2.16 ± 0.16	2.16 ± 0.22	VDR [pmol/l]	I	23.88 ± 0.91	24.44 ± 0.66
	II	2.24 ± 0.07	2.26 ± 0.11		II	24.17 ± 0.66	24.07 ± 0.71
	III	2.23 ± 0.06	2.19 ± 0.35		III	23.98 ± 0.48	23.90 ± 1.69
	IV	2.23 ± 0.05	2.30 ± 0.10		IV	24.30 ± 0.69	23.78 ± 0.65

TG—Nordic walking group; CG—control group; VDR—vitamin D receptor. I—baseline; II—after 3 weeks; III—after 6 weeks; IV—after 9 weeks (follow up). *^,#,$^—significant differences in TG.

## Data Availability

Data are available upon request from the authors.
